# Peer review

**DOI:** 10.3402/jchimp.v2i3.19690

**Published:** 2012-10-15

**Authors:** Robert P. Ferguson, Stephanie M. Griffin

Peer review seems to have had its origins in 18th century England to combat plagiarism (1). But today, the objectives of peer review are to provide evaluations of the quality of scientific or editorial material submitted. Peer review provides a check on the validity of manuscripts and sets standards for scientific merit. Peer review should help editors in making decisions about the manuscripts by offering second and third opinions. It lifts the quality of published articles to a higher standard. Readers have greater respect for and confidence in peer-reviewed publications. The first question I was asked by a prospective author invited for JCHIMP, volume 1 #1, was ‘Is it a peer review journal?’.

Peer review should not only address the scientific merit and integrity of the article but also the appropriateness of the submitted material. It should help editors in deciding whether an article merits publication or whether it requires major or minor revision prior to publication. It should, most importantly, help the authors through constructive criticism. It should point out the strengths and limitations of an article. The code of conduct of peer review has been detailed by others (2).

An excellent and comprehensive CD-ROM from the American Journal of Emergency Medicine is a resource on peer review, especially for novice peer reviewers (3). The authors have organized a stepwise methodology for reviewing a manuscript. This article appropriately links the abstract with the rest of the manuscript. The authors point out that an abstract should be read first for clarity, followed by available tables and figures, and see if they tie in adequately with the abstract. The text should then be evaluated, detailed, and summarized. The reviewer should finally revisit the abstract to see if the abstract provides insight into what lies ahead.

## Training for peer review

For many years, there has been a call to provide more training for peer reviewers (4). In my experience as a new editor, novice peer reviewers who are interested in participating in this process are looking for help in the approach to peer review. They have asked me for advice on numerous occasions. Recently, we proposed a workshop on training peer reviewers for the Association of Program Directors in Internal Medicine semi-annual meeting but were turned down because the program planning committee felt that this is a basic skill that all program directors had already acquired. We would disagree.

We try to make peer reviewing a positive experience for reviewers – a form of faculty development. The qualities of a good peer review include someone who takes his or her time, but not too much time. Reviewers should be constructive and understand the process. The reviewer should consider the fact that many authors in the JCHIMP journal are from community hospitals that frequently have a majority of faculty and residents who do not speak English as their primary language (5). This is of importance, as a poorly written manuscript sets the review off on a bad tone for the reviewer. Consequently, we often have to deal on two levels: one, the quality of research, and two, the English skills.

## Reasons to peer review

Why should someone participate in peer review? First, it is fun. It is also very interesting. It improves the reading and writing skills of the reviewer and allows the reviewer to participate at a scientific level that he or she may not have done previously. It has a steep learning curve, as there is very little training for many academic internists in peer review. Being selected as a peer reviewer indicates respect within your field. In our journal, we publish the list of peer reviewers on an annual basis. A major factor in the development of JCHIMP was to get more community hospital faculty involved and acknowledged as peer reviewers. We also pledge not to overburden peer reviewers and typically do not ask a reviewer to review more than two to three times a year. In addition, peer reviewing makes a significant contribution to scholarship.

One thing I have noticed, having participated in the peer review of more than 80 manuscripts reviewed by 83 reviewers with 180 reviews over the last 18 months since the inception of the journal, is how important and contributory peer reviewing can be to IMPROVING articles. It not only improves the writing skills of authors but also the writing skills of the reviewers, making them better critics. It also improves the reviewers’ diplomatic skills. Some reviewers have demonstrated, in their review, an antagonistic tone if they are put off by language or grammar or if they feel put upon to do this. We strongly encourage reviewers to take a constructive tone. I know that in my first 18 months as the editor of JCHIMP, I have learned a great deal about writing (although the quality of my writing in this perspective may cast some doubt on that). My experience with novice reviewers is that they are looking for help and guidance. References cited in this article can be an excellent resource. Another point of emphasis that I try to communicate is that there are different standards for different types of manuscripts. We publish retrospective studies, case reports, personal perspectives, and historical reviews, as well as interesting EKGs and X-rays. Callaham's CD-ROM provides an excellent reference for prospective original research in all medical fields (1).

## Reviewing a case report

In writing a case report review, start with the summary of the report either from the opening paragraph or the abstract. In case reports, we are not necessarily looking for one-of-a-kind cases, but cases that, due to their unique presentation and/or historical depth, provide information that allows for better understanding of a medical problem (6). Case reports can provide in-depth understanding of clinical problems–exceptions that prove the rule. A good case report can be the ultimate in the clinical competency of practice-based learning. The value of reporting cases has been pointed out by many (6–8). Published case reports have largely become a thing of the past, as case reports do not generate ‘academic credit’ for journals since they are infrequently cited. The hallmark of a good case report presentation is one that leads to clarity of clinical insights. If the reviewer is nodding his or her head with a smile while reading the case and remembering, the case reporting is justified.

## Blinded manuscripts

We believe it is very important for peer reviewers to be blinded to the origin of the manuscript. All the reviewers in JCHIMP are blinded and do not know the institutional source of the article. This is a key point because in my experience as a scientific reviewer, an article that comes out of certain institutions is looked at quite differently from an institution of renown rather than a program not known to the reviewer.

## Suspicions of plagiarism

It is permitted for the reviewer to raise questions about plagiarism. Plagiarism may occur more frequently in manuscripts submitted by authors whose primary language is not English and who may not understand the plagiaristic standards in the United States and find that using other people's sentences improves their article (1). One of the suggestions we have made for reviewers, who suspect part of the article may be plagiarized, particularly certain sentences or paragraphs standing out from the rest, is to ‘Google’ the words in question. We used this recently when a sentence was stylistically much different (better) than those written in the rest of the article. When the reviewer googled the sentence, he found an identical sentence in a case report from 10 years earlier from Canada. Because this is not necessarily intentional or may be the result of a misunderstanding, it should lead to counseling the author appropriately.

## Timing of review report

The peer review process should be timely. If the reviewer does not have time or feels that the manuscript is out of his depth, the editor should be notified as soon as possible. It is understandable given the hectic nature of all our schedules. One of the advantages of electronic media publications, such as JCHIMP and other internet based journals, is the efficiency with which submitting manuscripts can reach publication. We average approximately 4 months from the time the manuscript is submitted to the time it is published. We recommended strongly that the reviewer should let the editor know within days if he or she is not able to spend time or has a lack of expertise to do an adequate review.

## Review style and tracking

In writing a peer review, it is best to summarize the manuscript initially and then give the details about specific suggestions including asking for more or less data. We do not encourage reviewers to ask for expanded discussions regarding tangential issues as this often makes a case report too wordy, exceeding the word limit, and delves into areas that are not relevant to the report.

Consider using technology to help in the review such as ‘tracking’. We have attached a table to get started with those unfamiliar with this method. (See addendum) Tracking is an easily learned skill that often makes life better for the reviewer and author. I find it remarkable as to how a well-tracked manuscript is often made better with suggestions and is often very well received by the author.

The reviewers should not participate if they suspect they know the manuscript's source. There should be no derogatory comments to the authors. One reviewer, for example, commented that the manuscript had a ‘sixth-grade level of writing’. Poor writing is a valid criticism but should be addressed separately to the editor. We have also included an attachment of forms that are recommended by our publisher in the overall evaluating the quality of the peer review.

All in all, peer review serves an important function. It is a positive experience for the reviewers if done correctly. It is a very positive experience for the authors and allows them to improve the manuscripts.

Elsewhere in this issue are seven manuscripts. The history of oral hypoglycemic agents compliments the history of insulin perspective in the previous issue (9). There is also a charming perspective entitled ‘Am I losing it?’ by a nonagenarian internist who doesn't miss a thing (10). There are three case reports: Cryptococcal brain abscesses mimicking other ring enhancing lesions (11), a case of hemolytic uremic syndrome rarely associated with C. difficile colitis (12); superior vena cava syndrome associated with a pacemaker (13). In addition, our radiology-imaging column has dramatic images related to endocarditis (14) and ECG images show a case of a related bundle branch block at a heart rate of 60 (15).

*Robert P. Ferguson, MD* Chief of Medicine*Stephanie M. Griffin, BBA* Fellowship Coordinator

## Addendum

Technology has dramatically changed the way we edit/review any piece of writing. One of the most amazing new tools is a creation from Microsoft-tracking. Tracking began in Microsoft 2003, but has recently become popular with administrative assistants, students, editors, etc. This tool allows you, as the reviewer, to make changes to a piece of writing and show the author what changes were made, e.g., deleting a sentence or adding punctuation marks that were missed. It also allows the reviewer to add comments in the form of ‘balloons’ right at the location of the manuscript referred to. This may be very helpful to the author; this way they can understand the reviewer's point of view.

There are many different capabilities with tracking such as delete, insert and comment. To start editing using tracking, go to the ***review tab*** and click ***track changes***. As soon as you click this, changes can be made and seen. Next to the track changes button there is a ***balloon option***. This help to show the edits made. There are three different options: the first is to show all changes in a ***balloon***; the second shows all changes ***inline*** (no balloons addition and deletion shown in the piece of writing); and third shows changes ***inline and the comments in balloons***. If you choose to change the way the edit appears, click the ***down arrow*** on track changes and click change ***tracking options***.

To ***add or delete*** changes the person editing will just click the part of the writing where the change needs to be made and make the adjustments. As a reviewer you may want to add a comment to the author or the editor, this can be done easily. Under the same ***review tab*** the reviewer would click the ***new comments buttons***. There are two ways to display this to the author/editor. To change the appearance, the reviewer would click the ***balloons button*** located in the same tab. To display all comments in balloons, click ***show only comments and formatting in balloons***. Another way to show comments, is to click ***show all revisions inline***. All comments will be made in a list that will appear on the left side of the screen.

After revisions have been made you may want to ***hide certain changes/comments*** or only s***how changes/comments*** to the author or editor. This can be done in a few easy steps. When hiding changes/comments, first select the ***review tab*** and in the tracking section click on ***show markup***. Next, you will see a ***drop down box*** that will give you choices on what you wish to be displayed and how it will appear. For example, you may choose to not show comments. ***Unclick your selection*** and it will then be hidden; to ***show your selection again just click*** and it will reappear in the document. Under the show markup button there is also an option that you can choose which reviewer's changes/comments you would like to view. Just as you did with comments you just click any or all reviews to show or hide their changes/comments.
